# Characterization of respiratory microbial dysbiosis in hospitalized COVID-19 patients

**DOI:** 10.1038/s41421-021-00257-2

**Published:** 2021-04-13

**Authors:** Huanzi Zhong, Yanqun Wang, Zhun Shi, Lu Zhang, Huahui Ren, Weiqun He, Zhaoyong Zhang, Airu Zhu, Jingxian Zhao, Fei Xiao, Fangming Yang, Tianzhu Liang, Feng Ye, Bei Zhong, Shicong Ruan, Mian Gan, Jiahui Zhu, Fang Li, Fuqiang Li, Daxi Wang, Jiandong Li, Peidi Ren, Shida Zhu, Huanming Yang, Jian Wang, Karsten Kristiansen, Hein Min Tun, Weijun Chen, Nanshan Zhong, Xun Xu, Yi-min Li, Junhua Li, Jincun Zhao

**Affiliations:** 1 BGI-Shenzhen, Shenzhen, 518083 China; 2 Laboratory of Genomics and Molecular Biomedicine, Department of Biology, University of Copenhagen, 2100 Copenhagen, Denmark; 3 State Key Laboratory of Respiratory Disease, National Clinical Research Center for Respiratory Disease, Guangzhou Institute of Respiratory Health, the First Affiliated Hospital of Guangzhou Medical University, Guangzhou, Guangdong 510120 China; 4 Institute of Infectious disease, Guangzhou Eighth People’s Hospital of Guangzhou Medical University, Guangzhou, Guangdong 510060 China; 5 Guangzhou Customs District Technology Center, Guangzhou, 510700 China; 6 Department of Infectious Diseases, Guangdong Provincial Key Laboratory of Biomedical Imaging, Guangdong Provincial Engineering Research Center of Molecular Imaging, The Fifth Affiliated Hospital, Sun Yat-sen University, Zhuhai, Guangdong 519000 China; 7 School of Future Technology, University of Chinese Academy of Sciences, Beijing, 101408 China; 8 Shenzhen Key Laboratory of Unknown Pathogen Identification, BGI-Shenzhen, Shenzhen, 518083 China; 9 The Sixth Affiliated Hospital of Guangzhou Medical University, Qingyuan People’s Hospital, Qingyuan, Guangdong China; 10 Yangjiang People’s Hospital, Yangjiang, Guangdong China; 11 State Key Laboratory of Bioelectronics, School of Biological Science and Medical Engineering, Southeast University, Nanjing, 210096 China; 12 Guangdong Provincial Key Laboratory of Human Disease Genomics, Shenzhen Key Laboratory of Genomics, BGI-Shenzhen, Shenzhen, 518083 China; 13 BGI Education Center, University of Chinese Academy of Sciences, Shenzhen, 518083 China; 14 Shenzhen Engineering Laboratory for Innovative Molecular Diagnostics, BGI-Shenzhen, Shenzhen, 518120 China; 15 James D. Watson Institute of Genome Science, Hangzhou, 310008 China; 16 Guangdong Provincial Academician Workstation of BGI Synthetic Genomics, BGI-Shenzhen, Shenzhen, 518120 China; 17 HKU-Pasteur Research Pole, School of Public Health, Li Ka Shing Faculty of Medicine, The University of Hong Kong, Hong Kong SAR, China; 18 BGI PathoGenesis Pharmaceutical Technology Co., Ltd., BGI-Shenzhen, Shenzhen, 518083 China; 19 Guangdong Provincial Key Laboratory of Genome Read and Write, BGI-Shenzhen, Shenzhen, 518120 China; 20 School of Biology and Biological Engineering, South China University of Technology, Guangzhou, China

**Keywords:** Bioinformatics, Gene expression profiling

## Abstract

Severe acute respiratory syndrome coronavirus 2 (SARS-CoV-2) has caused a global pandemic of Coronavirus disease 2019 (COVID-19). However, the microbial composition of the respiratory tract and other infected tissues as well as their possible pathogenic contributions to varying degrees of disease severity in COVID-19 patients remain unclear. Between 27 January and 26 February 2020, serial clinical specimens (sputum, nasal and throat swab, anal swab and feces) were collected from a cohort of hospitalized COVID-19 patients, including 8 mildly and 15 severely ill patients in Guangdong province, China. Total RNA was extracted and ultra-deep metatranscriptomic sequencing was performed in combination with laboratory diagnostic assays. We identified distinct signatures of microbial dysbiosis among severely ill COVID-19 patients on broad spectrum antimicrobial therapy. Co-detection of other human respiratory viruses (including human alphaherpesvirus 1, rhinovirus B, and human orthopneumovirus) was demonstrated in 30.8% (4/13) of the severely ill patients, but not in any of the mildly affected patients. Notably, the predominant respiratory microbial taxa of severely ill patients were *Burkholderia cepacia* complex (BCC), *Staphylococcus epidermidis*, or *Mycoplasma spp*. (including *M. hominis* and *M. orale*). The presence of the former two bacterial taxa was also confirmed by clinical cultures of respiratory specimens (expectorated sputum or nasal secretions) in 23.1% (3/13) of the severe cases. Finally, a time-dependent, secondary infection of *B. cenocepacia* with expressions of multiple virulence genes was demonstrated in one severely ill patient, which might accelerate his disease deterioration and death occurring one month after ICU admission. Our findings point to SARS-CoV-2-related microbial dysbiosis and various antibiotic-resistant respiratory microbes/pathogens in hospitalized COVID-19 patients in relation to disease severity. Detection and tracking strategies are needed to prevent the spread of antimicrobial resistance, improve the treatment regimen and clinical outcomes of hospitalized, severely ill COVID-19 patients.

## Introduction

As of 31 January 2021, severe acute respiratory syndrome coronavirus 2 (SARS-CoV-2) has infected more than 102 million and resulted in more than 2.2 million deaths worldwide^1^. The pandemic poses a significant threat to public health and the global economy.

Respiratory viruses, such as coronaviruses and influenza virus, can lead to acute damage of the epithelial barrier and facilitate invasions of other pathogens^2,3^. For instance, secondary infections by *Stenotrophomonas maltophilia*, *Klebsiella pneumoniae*, or *Escherichia coli* were reported to cause serious complications in patients with SARS, such as bacteremia, sepsis, and nosocomial pneumonia (NP)^4^. In addition, *Streptococcus pneumoniae*, *Haemophilus influenzae*, and *Staphylococcus aureus* were frequently associated with NP and mortality in influenza pandemics^5^. It was estimated that approximately 29%–55% of the total 300,000 deaths in the 2009 H1N1 pandemic were caused by secondary bacterial NP^6–8^.

Concerns about the coinfections of SARS-CoV-2 with known viruses, bacteria, and fungi have also been raised. In severely ill patients, acute respiratory distress syndrome deteriorates patients’ conditions rapidly, and mechanical ventilation is generally required^9,10^. Such invasive procedures can further increase the risks of ventilator-associated pneumonia in these patients^11^. In 99 confirmed Wuhan patients enrolled in January 2020, one (1%) had positive cultures of *Acinetobacter baumannii*, *K. pneumoniae*, and *Aspergillus flavus*, and four (4%) were diagnosed with infection by *Candida*, but no influenza viruses were detected^9^. A later retrospective study in Wuhan patients further demonstrated that half of the deceased patients (27 out of 54) had experienced secondary infections^12^. By using real-time reverse transcriptase-polymerase chain reaction tests, Kim et al.^13^ recently reported a 20.7% (24 out of 116 specimens) coinfection rate with SARS-CoV-2 and other respiratory viruses in Northern California, including rhinovirus (6.9%) and orthopneumovirus (5.2%). However, microbial coinfections and their possible effects on clinical outcomes of SARS-CoV-2-infected patients remain largely unknown.

Here, combining diagnostic technologies (cultures and colorimetric assays) and metatranscriptomic sequencing, microbial coinfections in a Guangdong cohort of 23 patients hospitalized with SARS-CoV-2 infection were comprehensively evaluated. Our results revealed distinct differences in microbial composition between mildly and severely ill patients in both the respiratory and gastrointestinal tract. We further demonstrate that *Burkholderia cepacia* complex (BCC) bacteria, *Staphylococcus epidermidis* and *Mycoplasma spp*. were most related to opportunistic pathogens of the respiratory tract in severe cases, possibly exhibiting resistance towards multiple antibiotics, increasing the risk for prolonged intensive care unit (ICU) stay or even associated with increased mortality. By contrast, *Veillonella*, *Neisseria*, *Streptococcus*, and *Prevotella* were identified as the dominant active microbes in the respiratory tract of patients with mild symptoms, similar to what has been reported for healthy adults without infection^14^. Our findings demonstrate the value of metatranscriptomics for an unbiased evaluation of the respiratory microbiota associated with SARS-CoV-2 and provide useful information and suggestions regarding the adequate monitoring and management of the multidrug-resistant (MDR) bacteria in the COVID-19 pandemic.

## Results

### Demographic information of patients and clinical specimens used in the study

Twenty-three patients with COVID-19 hospitalized in the period 10 January–31 March 2020, in four hospitals in the Guangdong Province, China, were enrolled in this study. Fifteen infected patients (41–79 year old) admitted to the ICU and receiving mechanical ventilation were defined as having severe COVID-19, and the remaining eight patients (2–65 year old) were mild cases (Supplementary Table S1). Briefly, 95.7% of the patients (22 out of 23) received antiviral medications. All severe cases received broad-spectrum antibiotics to prevent and control nosocomial infections, and simultaneously 93.35% (14 out of 15) received antifungal agents (Supplementary Table S1). Also, 60% (9 out of 15) of severely ill patients received invasive mechanical ventilation. By contrast, none of the mild cases were treated with antibacterial or antifungal drugs. Up to 31 March, 2020, 53.3% (8 out of 15) of the severe ill patients had been transferred out of ICU or discharged from hospitals, and all mild cases had been discharged, whereas a 79-year-old patient died one month after admission to ICU (P01) (Supplementary Table S2). Sixty-seven serial clinical specimens from the respiratory tract (RT) (*n* = 47, sputum, nasal and throat swab) and gastrointestinal tract (GIT) (*n* = 20, anal swab and feces) of these patients were obtained between 27 January and 26 February 2020 for a comprehensive assessment of microbial characteristics after SARS-CoV-2 infection. A detailed timeline of specimen collections and clinical events for the 23 COVID-19 cases are shown in Supplementary Table S2.

### Workflow of ultra-deep metatranscriptomic sequencing

After quality control, an average of 268.3 Gb metatranscriptomic data were generated per sample (Supplementary Table S3). We applied an integrated bioinformatics pipeline to detect human, viral, and nonviral microbial reads in the total RNA-seq data (Supplementary Fig. S1 and Materials and methods). The percentage of human RNA reads (including human rRNA and non-rRNA human transcripts) varied between different types of specimens, constituting a high fraction of total reads among RT specimens (average percentage of 64.56%) and a low fraction among GIT specimens (average percentage of 22.56%) (Supplementary Table S3 and Fig. S1). After removing host data, SortMeRNA was applied^15^ to filter microbial rRNA from the metatranscriptomic data. The final remaining nonhuman nonmicrobial rRNA data (ranged from 386 Mb to 145 Gb) were then used to assess viral and nonviral microbial composition by Kraken2X^16^ and MetaPhlAn2^17^, respectively (Materials and methods). Detailed data statistics for each processing step are provided in Supplementary Table S3.

### Co-detection of viruses in clinical specimens of COVID-19 patients

**Fig. 1:**
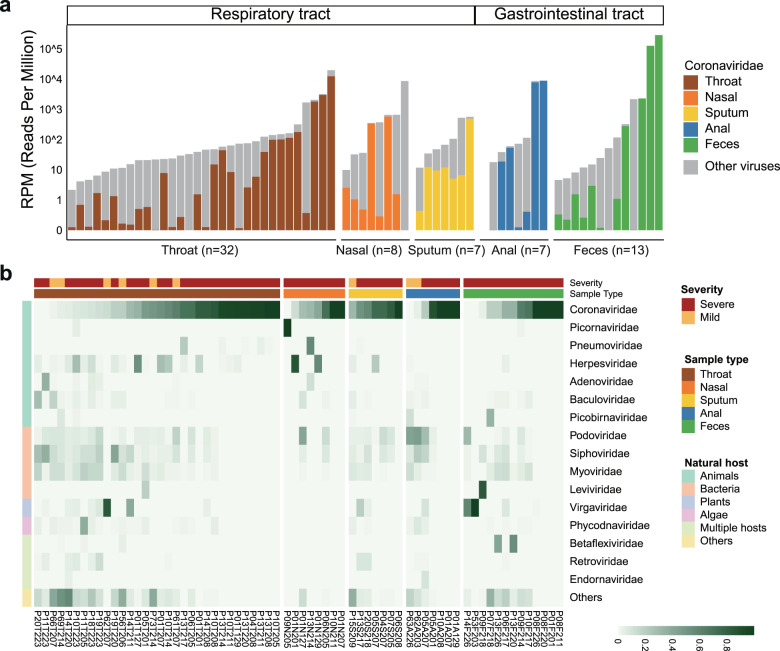
Viral RNA profiles in clinical specimens of hospitalized patients with COVID-19. **a** Bar plot showing the number of total viral reads and Coronaviridae reads in 67 clinical specimens collected from the respiratory and gastrointestinal tract. Data have been normalized to total sequencing reads in reads-per-million (RPM). The Coronaviridae reads of different sample types are colored as follows: brown, throat swab; orange, nasal swab; yellow, sputum; blue, anal swab; green, feces. Gray, non-Coronaviridae viral reads. **b** Heatmap showing the viral RNA relative abundance at the family level. Top 16 viral families are shown and ranked according to their natural hosts: green, animals; pink, bacteria; light blue, plant; purple, algae; light green, multiple host species; yellow, others (viral families of low abundances). Specimens from patients with mild and severe COVID-19 symptoms are colored by brick red and orange, respectively.

We first assessed the viral composition in RT and GIT specimens. As expected, *Coronaviridae* (mostly contributed by reads assigned to SARS-related coronavirus, Supplementary Table S3) was the most abundant virus, and was detected in all clinical specimens and varied between 0.01 and 286,418 mapped reads count per million (RPM) (Fig. 1a). Given the presence of confounding factors including days post symptom onset and various treatments on severe cases^18^, no comparison of the temporal abundance of SARS-CoV-2-like virus was conducted between mild and severe groups or between types of specimens. Although the SARS-CoV-2 RPM in samples of RT and GIT decreased consistently at later time points of infection (Supplementary Fig. S2a, b), it varied in different severely ill patients. For instance, RT specimens had consistently lower SARS-CoV-2 RPM than GIT specimens in P01, while specimens from the two sites showed comparable viral levels in P05 and P10 across all sampled time points (Supplementary Fig. S2c).

Besides *Coronaviridae*, RNA-seq analysis also revealed a great diversity of viral composition in clinical samples from infected patients. Natural hosts of the highly abundant viruses differed, including but not limited to animals (e.g., *Picornaviridae*, *Pneumoviridae*, and *Herpesviridae*), bacteria (e.g., *Podoviridae*, *Siphoviridae*, and *Myoviridae*), and plants (*Virgaviridae*) (Fig. 1b and Supplementary Table S4). The co-detection of other high-titer, known human respiratory viruses (genome coverage for representative viral genomes > 50%) was further confirmed in four out of thirteen severely ill patients with metatranscriptomic data of samples from the respiratory tract (30.8%), including human alphaherpesvirus 1 in P01 and P05, rhinovirus B in P09, and human orthopneumovirus in P13 (Fig. 1b and Supplementary Fig. S3a, b). The changes in relative abundance (presented in the unit RPM) of human alphaherpesvirus 1 and human orthopneumovirus in throat samples of P01 and P13 were similar to that of SARS-CoV-2 in the same patient (Supplementary Fig. S3c, d). Although some case studies reported the coinfection of SARS-CoV-2 and influenza viruses^19,20^, this was not observed in this Guangdong cohort. By contrast, none of these common human respiratory viruses were consistently detected in mild cases without ICU admission (Supplementary Table S4).

**Fig. 2:**
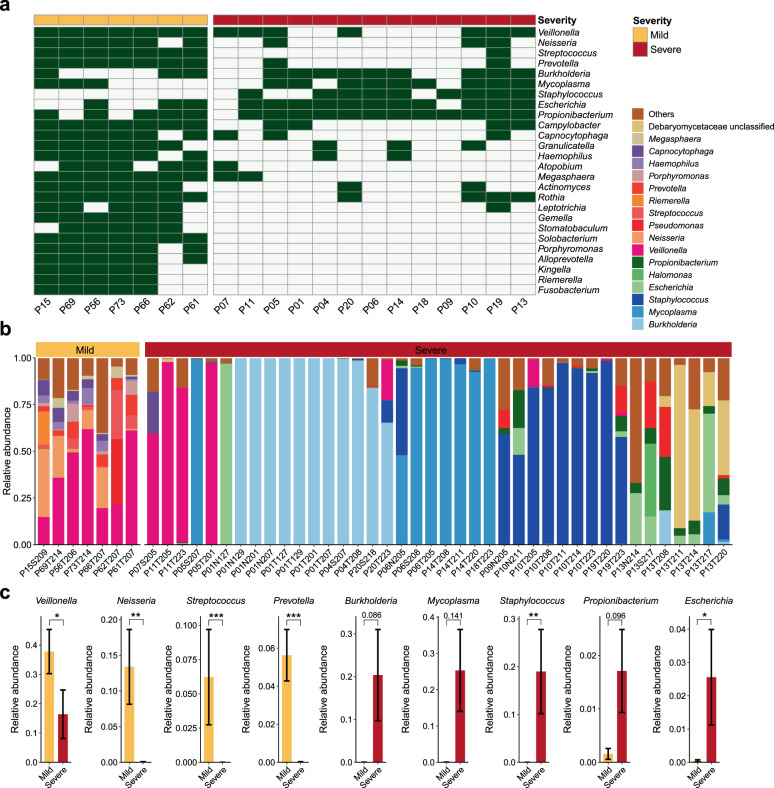
Distinct respiratory microbial signatures in mild and severe cases. **a** Presence/absence profile of nonviral microbial genera in mild and severe cases. Orange, mild; brick red, severe. Only common genera detected in over 60% of patients in the mild cases (*n* > 4) or severe cases (*n* > 7) are shown. **b** Bar plot showing the relative expression levels of nonviral microbes in all respiratory specimens of mild (orange, *n* = 7) and severe cases (brick red, *n* = 40). **c** Relative expression levels of selected genera differing between mild and severe cases. The bar chart and black error bars denote the mean and standard error values of expression levels in mild (orange) and severe (brick red) cases for each genus. ***P < 0.001; **P < 0.01; *P < 0.05; Wilcoxon rank-sum test. For patients with multiple respiratory specimens (all were severe cases), the presence of a given genus is considered when at least one sample from this patient was positive for the taxon (relative abundance > 0) (**a**), and the comparisons between relative expression levels of selected genera are conducted across all collected respiratory samples between mild and severe cases (**c**).

Additionally, plant viruses belonging to the family *Virgaviridae*, especially Pepper mild mottle virus (PMMoV) and Tomato mosaic virus (ToMV), were found to be the most dominant viruses in two fecal specimens (P53F203 and P14F226) (Fig. 2b and Supplementary Table S4). Although some pioneer studies have also reported strong evidence supporting the presence of PMMoV and ToMV in human-associated samples^21–23^, the presence of plant viruses in the fecal samples might also be obtained from food. The extent of possible virus transmission between plants and humans or other vertebrates remains largely unknown.

### Characterization of microbial dysbiosis in clinical specimens of COVID-19 patients

We next analyzed the hospital-laboratory-based results as well as the metatranscriptomic sequencing-based nonviral microbial composition to identify key active bacterial/fungal members that might be associated with clinical outcomes in hospitalized patients. Notably, results of cultures and laboratory assays on clinical specimens demonstrated the presence of potential nosocomial fungal (*n* = 1) and bacterial coinfections (*n* = 3) in severely ill COVID-19 patients (Supplementary Table S1). In detail, one patient (P01) tested positive for (1–3)-β-d-glucan (a common component of the fungal cell wall) in blood samples. Two patients (P04 and P20) had positive sputum cultures for *Burkholderia cepacia* complex (BCC) species, the most common respiratory pathogens causing NP in cystic fibrosis (CF) patients^24,25^. *S. epidermidis*, a typical skin bacterium that has been increasingly recognized as a MDR nosocomial pathogen^26,27^, was identified by culturing multiple nasal secretions of one patient (P06).

Next, the nonviral RNA data of all 67 clinical specimens were analyzed to fully assess the active microbial composition using MetaPhlAn2. As none of the mild cases were admitted to ICU or received antibacterial/antifungal agents, we compared the RT specimens between mild (*n* = 7) and severe (*n* = 13) cases to examine the microbial dysbiosis in patients exhibiting differential disease severity. Remarkable differences in RT microbial richness (number of detected taxa) and composition between mild and severe cases were observed (Fig. 2). All RT specimens (including six throat swabs and one sputum) of mildly ill patients who were admitted to three hospitals (located in Guangzhou, Yangjiang and Qingyuan, Supplementary Table S1) consistently exhibited a larger number of detected microbial taxa (Fig. 2a and Supplementary Fig. S4a) and similar microbial RNA community compositions (Fig. 2b and Supplementary Fig. S4b). Notably, the number of respiratory microbial taxa (at the genus and species level) detected in mild cases was significantly higher than that in severe cases (Supplementary Fig. S4c, *P* < 0.001, Wilcoxon rank-sum test). On average, 28 genera and 48 species were detected per RT sample in mild cases, while only six genera and four species were detected per RT sample in severe cases (Supplementary Fig. S4c and Table S7), which might reflect the pronounced effects of the administration of broad-spectrum antimicrobial agents in these severely ill patients. The predominant RT bacteria in mild cases were *Veillonella*, *Neisseria*, *Streptococcus*, and *Prevotella* (occurrence > 80% individuals and mean relative abundance > 5%) (Fig. 2b, c), which is similar to microbial communities commonly reported in the nasal and oral cavity of healthy human adults^14,28^. However, except for *Veillonella*, each of the latter three genera enriched in mild cases was only detected in few severe cases (*n* ≤ 3, Fig. 2a). The four genera associated with mild cases also showed significantly higher mean abundance in mild than in severe cases (Fig. 2c, *P* < 0.05, Wilcoxon rank-sum test).

Of note, several prevalent RT microbial features in severe cases were identified to be patient-specific. Among 40 respiratory samples from severe patients, over 60% were mono-dominated (relative abundance > 60%, as suggested by Hildebrandt et al.^29^) by the bacterial genus *Burkholderia* (11 samples from P01, P04, and P20), *Staphylococcus* (6 samples from P10 and P19) or *Mycoplasma* (7 samples from P05, P06, P14, and P18) (Fig. 2b, c and Supplementary Table S2). Each genus was detected in 69.2% of (9 out of 13) severely ill patients, and 92.3% (12 out of 13) of severely ill patients were positive for at least one of the three genera (Fig. 2a), indicating their prevalence in RT of patients hospitalized with severe COVID-19 symptoms. By contrast, positive detection of *Staphylococcus* RNA reads was not observed in any RT samples from mild cases (Fig. 2a). Therefore, the presence of *Staphylococcus* could hardly be considered as contaminants from sampling, hospital environment, or reagents for RNA extraction and sequencing, as the experimental procedures were performed simultaneously at the same time on all clinical specimens from both mild and severe cases to minimize the batch effects and possible microbial contaminations. By mapping RNA reads to the reference genomes of BCC species (Materials and methods), we further confirmed the predominant expression of *B. cenocepacia* in the respiratory tract of P01, and *B. multivorans* in P04 and P20, who also provided positive sputum cultures of BCC (Supplementary Fig. S5a and Table S8). All *Staphylococcus* RNA reads of RT samples from P06 (who also provided positive *S. epidermidis* culture), P10 and P19 were assigned to *S. epidermidis* (Fig. 2b and Supplementary Fig. S5b). However, *S. aureus*, a major hospital-acquired pathogen^30^, was not detected in metatranscriptomic data of any sequenced RT samples. *Mycoplasma orale* and *M. hominis*, rather than *M. pneumoniae*, were the two dominating *Mycoplasma* members (Fig. 2b and Supplementary Fig. S5c). *Propionibacterium* and *Escherichia* were also frequently detected in RT samples from severe cases (occurrence > 80% individuals) but were less abundant than the former three genera (mean relative abundance < 3%) (Fig. 2c). Moreover, all the five prevalent genera in severe cases have been reported to be antibiotic-resistant bacteria and/or associated with nosocomial infections, while they were not detected or present in extremely low abundance in mild cases (relative abundance < 0.15%) (Fig. 2a, c and Supplementary Table S6).

**Fig. 3:**
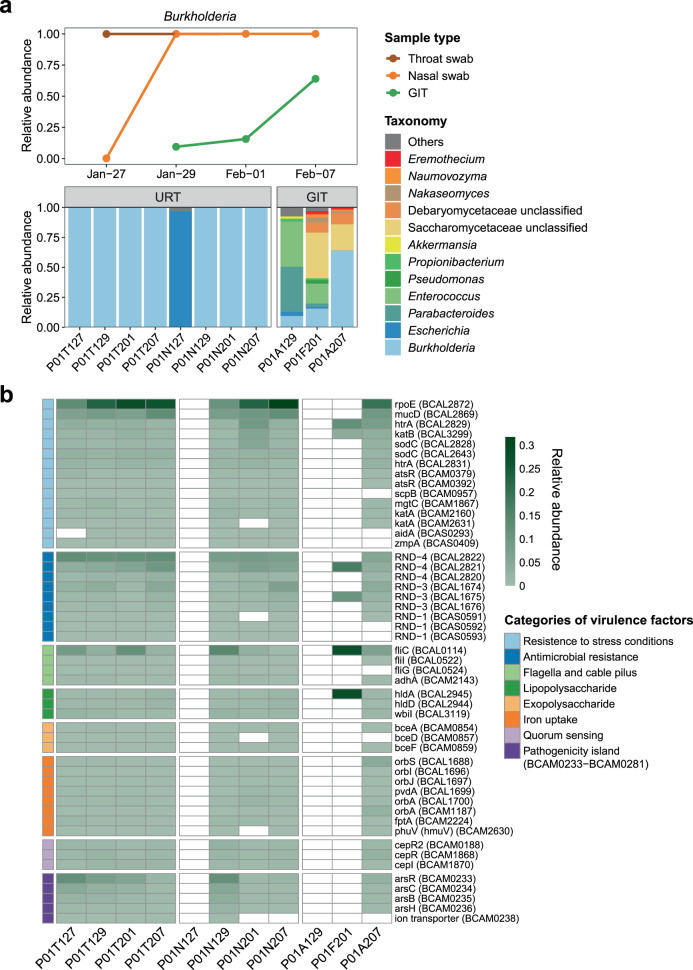
Identification of the potential secondary *B. cenocepacia* infection in P01. **a** Bar plot showing the relative expression levels of nonviral microbes in all specimens from P01. A timeline chart showing the corresponding changes in abundance of *B. cenocepacia* transcripts from 27 January to 07 February 2020 in P01. Brown indicates throat swabs; orange indicates nasal swabs and green indicates GIT samples including anal swabs and feces. A total of 11 specimens from the respiratory tract (RT) and gastrointestinal tract (GIT) are shown. Orange, fungi; blue, bacteria. **b** Heatmap showing the relative expression levels of virulence factors of *B. cenocepacia*. A total of 50 identified virulence genes are shown and ranked according to their functional categories: light blue, resistance to stress conditions; blue, antimicrobial resistance; light green, flagella and cable pilus; green, lipopolysaccharide; pink, exopolysaccharide; orange, iron uptake; light purple, quorum sensing; purple, genes located in a pathogenicity island.

Consistently, we detected both positive results for the blood (1–3)-β-d-glucan levels (Supplementary Table S9) as well as GIT expression of ascomycetic transcripts (mainly from the genus *Saccharomycetaceae*) in the P01 (Supplementary Table S6 and Fig. 3a), who died one month after ICU admission. Ascomycetic transcripts (mainly from *Debaryomycetacea*) were also identified in all five throat swabs collected from P13 (Fig. 2b). Interestingly, the ascomycetes constituted only 5.7% of the total nonviral microbes at the first time point (8 February) and increased to 18.2%–87.4% at later points (11-20 February) (Fig. 2b). P13, a 79-year-old man, had onset of COVID-19 symptoms on 30 January and had been admitted to the ICU since 5 February (Supplementary Table S1). Although the patient received daily antimicrobial treatment with a combination of antibiotic (meropenem, targocid, polymyxin b sulfate, amikacin, or sulperazone), antifungal (cancidas and/or amphotericin B) and antiviral drugs (ribavirin) during the entire sampling period (6 February to 26 February), these observations suggested that a rapid succession from bacteria to fungi had occurred in the microbiota of the respiratory tract in P13 three days after ICU admission. These findings collectively indicated that serial monitoring to track respiratory fungi and possibly secondary fungal infections is required to avoid delayed treatment for such patients.

In addition, three severe cases (P05, P07, and P11), despite receiving multi-agent antimicrobial therapy (Supplementary Table S1), had high expression levels of *Veillonella* but low levels (or no detection) of the above potential opportunistic pathogens (or pathogens) in their RT samples (Fig. 2a, b). Most well-known respiratory bacterial pathogens, as well as potential high-abundant pathogenic candidates we identified/isolated in clinical specimens of COVID-19 patients with severe symptoms, are aerobic or facultative organisms. In contrast, *Veillonella spp*. are strictly anaerobic and have been reported to be part of normal oral cavities and rarely isolated in nosocomial infections^31,32^. Of note, all three patients (P05, P07, and P11) had been transferred out of the ICU or discharged from hospitals and none of them received invasive ventilation during ICU admission (Supplementary Table S1).

In addition, severe cases also appeared to have a distinct gut metatranscriptome compared to mild cases. GIT specimens (anal swabs) from the two mild patients with no antimicrobial treatments consistently showed high abundances of Proteobacteria (e.g., *Campylobacter*) and *Streptococcus* in their gut metatranscriptome (Supplementary Fig. S6a). Similarly, a recent study using 16S rRNA gene-based amplicon sequencing (fecal samples) also reported a significantly higher relative abundance of *Streptococcus* in antibiotic treatment-naïve COVID-19 patients than in age-, sex-, and body mass index-matched healthy controls^33^. Our mild case-related GIT microbial transcripts using anal swabs also differed from the previous metatranscriptomic study of healthy adults using fecal samples, whose gut microbiota was dominated by Firmicutes and Bacteroidetes^34^. Longitudinal samples from both patients and healthy controls are needed to characterize the COVID-19-related gut microbial dysbiosis using the same sampling sites and sequencing strategy.

On the other hand, *Parabacteroides* constituted one of the major active GIT bacteria of the severe cases contrasting mild cases. For instance, *Parabacteroides* (including *P. distasonis* and *P. merdae*) mono-dominated the gut microbial transcripts in five fecal samples from four severe cases (relative abundance > 60%, P07, P09, P10, and P13) (Supplementary Fig. S6b–d). The genus also displayed a relatively high abundance in several other fecal samples and anal swabs of severe cases (relative abundance > 20%) (Supplementary Table S8). Interestingly, an extreme bloom of *P. distasonis*, a low-abundant but common taxa in the human gut, has been reported after beta-lactam ceftriaxone treatment^29^. Thus, metatranscriptomic findings have not only complemented and enhanced the laboratory-based detection of candidate pathogens but also provided comprehensive information on microbial dysbiosis in COVID-19 patients.

### Identification of secondary coinfection with *B. cenocepacia*

In order to retrospectively investigate possible non-COVID-19 risk factors associated with the in-hospital death of patient P01, serial clinical specimens were collected and analyzed. Notably, a distinct co-detection of transcripts belonging to *B. cenocepacia* was clearly observed in a time-dependent manner in this patient. On the first day of sampling (27 January 2020), up to 99.9% of nonviral microbial transcripts in his throat swab were assigned to *B. cenocepacia* (P01T127), while most of the transcripts in his nasal swab collected on the same day were from *Escherichia* (mainly from *E. coli*) (P01N127) (Fig. 3a). *B. cenocepacia* was subsequently predominantly present in both throat and nasal swabs for all the following sampling time points (29 January–07 February 2020) (Fig. 3a). In addition, *B. cenocepacia* was detected in all GIT samples from this patient, and the percentage of *B. cenocepacia* to nonviral transcripts gradually increased from 9.5% to 64% (from 29 January to 7 February) (Fig. 3a). The time-dependent dynamics of transcript levels of *B. cenocepacia* suggested that transfer from the upper respiratory tract to the lower gastrointestinal tract had caused a secondary systemic infection in P01. Our findings were also consistent with the patient’s death certificate record, indicating bacteremic sepsis as one of the leading causes of his death (Supplementary Table S1). Indeed, several retrospective studies have pointed out that among BCC-infected CF patients, infection with *B. cenocepacia*, rather than other commonly isolated BCC members (such as *B. multivorans* and *B. cepacia*), constituted the highest risk factor of death^25,35^.

Next, virulence factors (VF) expressed by *B. cenocepacia* in P01 were analyzed to understand better the pathogenic mechanisms of this possible lethal pathogen in this severe COVID-19 case (Materials and methods). The gene *rpoE* (a member of the extracytoplasmic function subfamily of sigma factors) was the most abundantly expressed VF during the entire sampling period in P01 with SARS-CoV-2 infection (Fig. 3b). RpoE, as a stress response regulator, has been demonstrated to be essential for the growth of *B. cenocepacia* and the delay of phagolysosomal fusion in macrophages during infection^36^. A delay in phagolysosomal fusion has also been an important host immune escape strategy for several bacterial pathogens^37^. Other VFs in response to oxidative stress conditions in the host environment, such as those encoding superoxide dismutase, peroxidase or catalase (*sodC* and *katB*) were also expressed (Fig. 3b and Supplementary Table S9). A panel of genes belonging to resistance-nodulation-division (RND) family transporters that confer multidrug resistance to *B. cenocepacia*^38^ were also highly expressed in all types of specimens. We also detected expressions of genes encoding flagella and cable pilus (*fliC*, *flil*, *fliG*, and *adhA*), which can facilitate the bacterial adhesion to host cells and mucin^39^ (Fig. 3b). In addition, expressions of genes involved in quorum sensing, iron uptake (by competing with the host for iron), biosynthesis of lipopolysaccharide (LPS) and exopolysaccharide (EPS) were also detected (Fig. 3b), indicating their active roles in the regulation of bacterial cell aggregation, biofilm formation and toxin production during infection.

## Discussion

In this study, ultra-deep metatranscriptomic sequencing combined with clinical laboratory diagnosis, including cultures and colorimetric assays, identified key characteristics of the microbial dysbiosis associated with hospitalized patients infected with SARS-CoV-2. The most prevalent respiratory bacteria in our severely ill COVID-19 patients were BCC bacteria, *S. epidermidis* and *Mycoplasma spp*. (including *M. hominis* and *M. orale*). These organisms are distinct from prior results on pathogenic bacteria identified in previous coronavirus outbreak and influenza pandemics (e.g., *S. pneumoniae*, *S. aureus*, *K. pneumoniae*, and *M. pneumoniae*)^4–8^ or those associated with ICU-acquired bloodstream infections (BSIs) (*Acinetobacter baumannii*, *K. pneumoniae*, *Enterococcus spp*., *Candida albicans*, and *C. parapsilosis*) in a cohort of 50 severely ill COVID-19 patients in Athens, Greece^40^. A strong confounding factor in this study is the markedly different treatment instigated for the mild and severe cases, where multiple and broad-spectrum antimicrobial agents were given to severe cases but not mild cases. Another limitation, however, is the relatively small sample size of patients (*n* = 23) as well as the highly biased number of specimens from severe and mild patients (58 vs 9). Also, uninfected controls were not enrolled in the study, though the microbial composition of the respiratory and gastrointestinal tract has been reported in healthy individuals^14,28,34^. All these inherent limitations, therefore, prevented our microbial observations in treatment-naïve mildly ill patients from being associated with COVID-19 infection, but provided important information of microbial dysbiosis and related multidrug resistance in severely ill patients receiving antimicrobial treatments.

In particular, respiratory *BCC* mono-dominated 23.1% of severe cases (relative abundance > 60%), showing co-detection evidence from both laboratory cultures and metatranscriptomic results in P04 and P20. The serial metatranscriptomic data of all specimens from P01 revealed the timeline of secondary nosocomial infection with *B. cenocepacia* alongside the expression of various virulence genes, which could confer the abilities of the lethal pathogen to evade host defenses (e.g., *rpoE*), adhere target tissues (e.g., flagella-coding genes and *adhA*), produce toxins (e.g., genes encoding biosynthetic enzymes for the production of LPS and EPS), and resist the effects of multiple antibiotics (RND family), which eventually may have led to the life-threatening bacteremic sepsis of the patient. In addition to our study, two recent studies also reported nosocomial BCC-associated deaths of COVID-19 patients^41,42^. However, all three studies are single case reports and could hardly be used to draw inferences about an undetected BCC-associated nosocomial outbreak whose primary sources have been identified to be contaminated medical products/devices (mainly the disinfectant products)^43^. BCC bacteria, a major threat to hospitalized CF patients, are predominantly localized in the phagocytes and mucus in the CF lung^44^ and might accelerate the decline in pulmonary function of COVID-19 patients. Although with a low reported incidence, given the lethal outcomes in relation to the BCC coinfections, management strategies should be developed for these hospitals to track the potentially infectious source of various medical products, the frequency of the infections occurred, and finally, to prevent nosocomial BCC-coinfections in hospitalized COVID-19 patients.

Except for *B. cenocepacia*, the other prevalent respiratory bacteria found in our severely ill patients (*B. multivorans*, *M. hominis*, *M. orale* and *S. epidermidis*) usually cause mild or no symptoms; however, they are reported to be widespread in hospital environments and may act as reservoirs for antibiotic resistance genes (ARGs)^45–47^. *Mycoplasma spp*. lack a cell wall and have inherent resistance to commonly administered beta-lactam antibiotics. Several studies further indicated escalating antibiotic resistance levels in mycoplasmas^45,46^, including macrolide, tetracycline, or fluoroquinolone classes of antibiotics. Super-high expression levels of *M. orale* genes were found in the RT of two severe ill patients (P14 and P18) with prolonged ICU stay (> 30 days), though both received antimicrobial therapy during this period. Similarly, among coagulase-negative staphylococci, *S. epidermidis* has been reported to cause the greatest number of nosocomial infections, particularly the chronic infections associated with indwelling medical devices^47^. Although S*. epidermidis* does not usually produce aggressive toxins, several hospital-adapted lineages have increasingly acquired clinically relevant resistance determinants and formed an unignorable challenge among nosocomial infections^27^. Additional evidence has demonstrated the horizontal transfer of ARGs, including methicillin resistance, from *S. epidermidis* to *S. aureus*^48–50^, a known formidable, virulent nosocomial pathogen. Our findings also agree with two independent studies that consistently reported a high incidence of ICU-acquired BSIs (51.2%–54%) in severely ill COVID-19 patients, mostly due to MDR pathogens^40,51^.

Currently, no effective drugs have been licensed for human use against SARS-CoV-2 infection. Instead, the widespread use of antimicrobial agents (including broad-spectrum antibiotics) has been documented in many studies (including ours)^9,12^ to prevent and treat possible secondary infections in COVID-19 patients, especially in those who needed mechanical ventilation. Without clinical specimens before any treatments and specimens from non-infected healthy controls, it is difficult to distinguish to what extent differences in the respiratory microbial patterns associated with SARS-CoV-2 infections reflect the disease or the antimicrobial treatment, or both. Still, the distinct bacterial communities as well as their dramatic and rapid shifts in the respiratory and gastrointestinal tract of the severely ill patients might be related to not only the excessive antimicrobial therapy in many cases but also the significant disruption of the normal human microbiota caused by the antimicrobial therapy allowing colonization by MDR organisms including opportunistic pathogens. Only a single patient died from secondary bacterial sepsis in our study, which recommends narrower and more targeted treatment therapy for secondary or coinfections with other organisms. Stepwise strategies are needed to monitor hospital/ICU acquired MDR organisms, control the spread of ARGs, optimize antimicrobial therapy for COVID-19 patients, and prevent potential future threats from a blooming reservoir of MDR organisms after the global pandemic, by using rapid diagnostic technologies such as antigen and antibody testing (e.g., immunofluorescence assays and enzyme-linked immunosorbent assays) and nucleic acid-based molecular testing (e.g., polymerase chain reaction and DNA microarray)^52^.

## Materials and methods

### Enrollment of hospitalized patients with SARS-CoV-2 infection, collection of clinical specimens

Twenty-three patients admitted to hospital in January 10–March 31, 2020, with confirmed SARS-CoV-2 infection based on a positive SARS-CoV-2 test were included. A total of 67 clinical specimens from the respiratory and gastrointestinal tract (including throat swab, nasal swab, sputum, anal swab and feces) were collected from the above patients at the First Affiliated Hospital of Guangzhou Medical University (thirteen patients), the Fifth Affiliated Hospital of Sun Yat-sen University (two patients), Yangjiang People’s Hospital (five patients) and Qingyuan People’s Hospital (three patients) between 27 January and 26 February 2020. All specimens were stored at −80 °C before nucleic acid extraction. Patients were classified into mild (*n* = 8, without intensive care unit admission) and severe (*n* = 15, with ICU admission) cases based on their severity of SARS-CoV-2 symptoms. Detailed de-identified information for patients and clinical specimens are presented in Supplementary Tables S1 and S2, respectively.

### Laboratory diagnosis of nosocomial bacterial and fungal infections

All severely ill COVID-19 patients admitted to the ICU for more than 48 h were monitored for nosocomial infections, which were defined according to the definitions of the US Centers for Disease Control and Prevention^53^. Culture of sputum and nasal secretions was conducted according to standard protocols for diagnosing nosocomial microbial infections (hospital-acquired and ventilator-associated) in all severely ill COVID-19 patients with ICU admission^54^. Blood samples of patients (P01 and P05) hospitalized in the Fifth Affiliated Hospital of Sun Yat-sen University were collected for colorimetric assay-based (1–3)-β-d-glucan test to diagnose fungal infection (Dynamiker Biotechnology, Tianjin, China, Catalog number: DNK-1401-1).

### RNA extraction, metatranscriptomic library preparation, and sequencing

For each clinical sample, total RNA was extracted (QiAamp RNeasy Mini Kit, Qiagen, Germany). DNA from human and microbes was then removed from RNA using DNase I and the concentration was quantified (Qubit RNA HS Assay Kit, Thermo Fisher Scientific, Waltham, MA, USA). Purified RNA samples were regularly shipped on dry ice to BGI-Shenzhen and subjected to preprocessing, DNA nanoball-based library construction, and high-throughput metatranscriptomic sequencing on the DNBSEQ-T7 platform (100 nt paired-end reads, MGI, Shenzhen, China)^55^. Three negative controls (NCs) from nulcease-free water were prepared for library construction and metatranscriptomic sequencing in parallel with the clinical samples as described previously in our polit study^55^ while all the NCs failed to yield any sequencing data due to relatively low biomass.

### Identification and removal of human RNA reads from metatranscriptomic data

For each sample, the raw metatranscriptomic reads were processed using Fastp (v0.19.5, default settings)^56^ to filter low-quality data and adapter contaminations and generate the clean reads for further analyses. Human-derived reads were identified with the following steps: (1) identification of human ribosomal RNA (rRNA) by aligning clean reads to human rRNA sequences (28S, 18S, 5.8S, 45S, 5S, U5 small nuclear RNA, as well as mitochondrial mt12S) using BWA-MEM (0.7.17-r1188)^57^; (2) identification of human transcripts by mapping reads to the hg19 reference genome using the RNA-seq aligner HISAT2 (version 2.1.0, default settings)^58^; and (3) a second-round identification of human reads by aligning remaining reads to hg 38 using Kraken2 (version 2.0.8-beta, default settings)^16^. All human RNA reads were then removed to generate qualified nonhuman RNA-seq data. The number of human RNA-seq reads identified at each step is presented in Supplementary Table S3.

### Characterization of viral communities in hospitalized patients with SARS-CoV-2 infection

Before the identification of virome and microbiota, SortMeRNA version 4.2.0^15^ (default settings) was applied to filter microbial rRNA (28S, 18S, 5.8S; and 23S, 16,and 5S rRNA from the SILVA database) from nonhuman metatranscriptomic data. Given the nucleotide-based methods exhibit lower accuracy on the viral read, we used Kraken2X which uses translated search against a protein database for viral classification^16^. The remaining nonhuman non-microbial rRNA reads were processed by Kraken2X v2.08 beta (default parameters)^16^ with a self-built viral protein database by extracting protein sequences from all complete viral genomes deposited in the NCBI RefSeq database (8872 genomes downloaded on 1 March 2020 including the SARS-CoV-2 complete genome reference sequence, GCF_009858895.2). The number of reads annotated to each viral family was summarized based on the read alignment results of Kraken2X. By ranking the number of *Coronaviridae* reads at the species level, we found that most of the *Coronaviridae* reads were annotated to SARS-related Coronavirus (Spearman’s rho > 0.996 between the number of RNA reads annotated to *Coronaviridae* and that annotated to SARS-related coronavirus) whereas only a tiny fraction of RNA reads (median number = 4) mapped to the common human coronaviruses (Human coronavirus NL63, 229E, and HKU1) (Supplementary Table S3). The remaining *Coronaviridae* reads were also mostly mapped to reference genomes of bat coronaviruses, which might result from a misclassification of SARS-related reads, as they are the closest relatives of SARS-CoV-2 (Supplementary Table S3). Based on the above observation, the Kraken2X-annotated *Coronaviridae* reads were considered as SARS-CoV-2-like reads in this study. For each sample, the ratio of SARS-CoV-2-like reads to total clean reads and the ratio of SARS-CoV-2-like reads to total viral reads were calculated accordingly (Supplementary Table S4).

After ranking the aligned reads of all detected viral species in each sample, highly abundant non-*Coronaviridae* viral species (> 10,000 aligned RNA reads per species) were identified (Supplementary Table S5) and selected for robust co-detection with known respiratory viruses in hospitalized patients with SARS-CoV-2 infection. Three human respiratory viral species (human alphaherpesvirus 1, human orthopneumovirus and rhinovirus B) co-detected in respiratory samples from four severe cases (P01, P05, P09, and P13) met the above criterion. Representative genomes of each species, including human herpesvirus 1 strain 17 (NC_001806.2), human orthopneumovirus subgroup A (NC_038235.1), and rhinovirus B isolate 3039 (KF958308.1) were downloaded from NCBI. One representative sample (P01N201, P05S207, P09N205, and P12T211) with the highest number of reads assigned to the targeted species was used for coverage analysis for each patient. Reads assigned to a given species were aligned against the corresponding reference genome by bowtie2 v2.3.0 (the ‘-sensitive’ mode, local alignment)^59^. Sequencing depth and genome coverage of each reference genome were determined with BEDTools v2.27.1 (genomecov -ibam sort.bam -bg)^60^. Robust co-detection with known respiratory viruses was defined when > 50% of the genome was covered. Considering the reported relatively low classification accuracy at the species level using the Kmer-based classification algorithm of Kraken, we did not focus on the possible low-titer, human viral species with low coverage and a small number of mapped reads.

### Characterization of nonviral microbial communities in hospitalized patients with SARS-CoV-2 infection

MetaPhlAn2, a clade-specific marker gene-based alignment method, requires considerable genome coverage and depth to detect marker genes and the presence of a given microbial taxon^17^. To minimize the detection of potential low-level microbial contaminants from environmental sources (e.g., reagents and kits), we used nonhuman non-rRNA reads (input RNA data) and MetaPhlAn2 (version 2.7.0) (default parameter options, except for–ignore-viruses)^17^. Importantly, the default parameter “stat-q” was set as 0.1^17^ and it excluded the 10% of markers with the highest and the 10% with the lowest abundance for calculating the robust abundance of a given taxon. The MetaPhlAn2-based relative abundance profiles of nonviral microbial taxa at the genus level were presented in Supplementary Table S6. The robust presence-absence profiles of respiratory microbial taxa at both genus and species levels are presented in Supplementary Table S7. Mono-dominance of a given microbial taxon (genus or species) was defined if a taxon had a relative abundance > 60% in one sample as suggested by a previous study^29^.

Most of the RNA reads of the two predominant bacterial genera *Burkholderia* and *Parabacteroides*, respectively, identified in the respiratory and gastrointestinal tract of severe cases could hardly be assigned to species level by MetaPhlAn2, which might reflect that the two genera contain closely related species that are difficult to differentiate by marker genes. In order to determine which species and how abundant species were in samples mono-dominated by *Burkholderia* or *Parabacteroides*, we downloaded four reference genome sequences of the most frequently isolated BCC species (*B. cenocepacia* J2315*, B. multivorans* ATCC BAA-247, *B. cepacia* ATCC 25416 and *B. dolosa* AU0158) and two gut *Parabacteroides* species (*P. distasonis* ATCC 8503 and *P. merdae* NCTC13052). For each sample, reads were mapped against corresponding references by bowtie2 v2.3.0, and the sequencing depth and genome coverage were estimated by BEDTools v2.27.1 as described above. The summary of coverage and depth of reference genomes for selected samples are presented in Supplementary Table S8. Prevalent SARS-CoV-2-associated respiratory bacteria or fungi were considered to be present if the respiratory specimens (at least one sample) of patients were mono-dominated (relative abundance > 60%) by a given taxon (metatranscriptomic sequencing) (Supplementary Table S9).

### Identification of expressed VF in *B. cenocepacia*

To identify the presence and expression patterns of potential VF in *B. cenocepacia* identified in P01, we collected multiple functional categories of virulence genes previously studied and verified by gene mutation analysis in *B. cenocepacia* strains as well as corresponding gene ID in the annotated J2315 genome^35^, including (1) resistance to stress conditions, (2) antimicrobial resistance, (3) quorum sensing, (4) iron uptake, (5) flagella and cable pilus, (6) LPS, and (7) EPS. In addition, a pathogenicity island identified on chromosome 2 (BCAM0233-BCAM0281) of *B. cenocepacia* J2315 by using comparative genomics was included^61^. Nonhuman non-rRNA microbial reads of all samples from P01 were mapped against the reference genome of *B. cenocepacia* J2315 using bowtie2 v2.3.0 as described above and identified by the gene IDs in the J2315 genome. For each sample, only virulence genes with more than 10 mapped reads were retained. A total of 50 expressed virulence genes were identified in clinical samples collected from P01 and presented in Supplementary Table S10.

To compare the expression levels between different genes, we performed normalization of target gene expression levels among all detected virulence genes using the following equation

$${{\rm{Gene}}\,{\rm{expression}}\,{\rm{level}}}_i = \frac{{\frac{{N_i}}{{Lg_i}}}}{{\mathop {\sum }\nolimits_{i = 1}^k \frac{{N_i}}{{Lg_i}}}}$$

where *i* (1, 2, … *k*) refers to a given virulence gene identified in *B. cenocepacia* J2315; *Lg*_*i*_ is the length of gene *i*; *N*_*i*_ is the reads number that mapped to gene *i*.

### Statistical analyses

Non-metric multidimensional scaling ordination of respiratory microbial community was conducted using the Manhattan distances based on a presence/absence matrix of genus profiles of 47 respiratory specimens (7 from mild cases and 40 from severe cases) (R version 3.6.1, vegan package). Wilcoxon rank-sum tests were performed to compare differences in the (mean) value of bacterial relative abundance at the genus level between the overall respiratory specimens of mild and severe cases, respectively (R version 3.6.1, coin package).

## Supplementary information

Supplementary Information (figures)

Supplementary Information (tables)

## Data Availability

The nonhuman metatranscriptomic sequencing data from 67 clinical specimens that support the findings of this study have been deposited into CNSA (CNGB Nucleotide Sequence Archive) of CNGBdb with accession number CNP0001066. Details of software, code, and parameters used for data analyses of the current study are provided and publicly available from GitHub.
