# Supplementary material for: Characterization of respiratory microbial dysbiosis in hospitalized COVID-19 patients
Source: Cell Discov. 2021 Apr 13;7:23. doi: 10.1038/s41421-021-00257-2 (PMC8043102; doi:10.1038/s41421-021-00257-2)
Supplement: Supplementary file 1 — Supplementary Information (figures) [file 41421_2021_257_MOESM1_ESM.pdf]

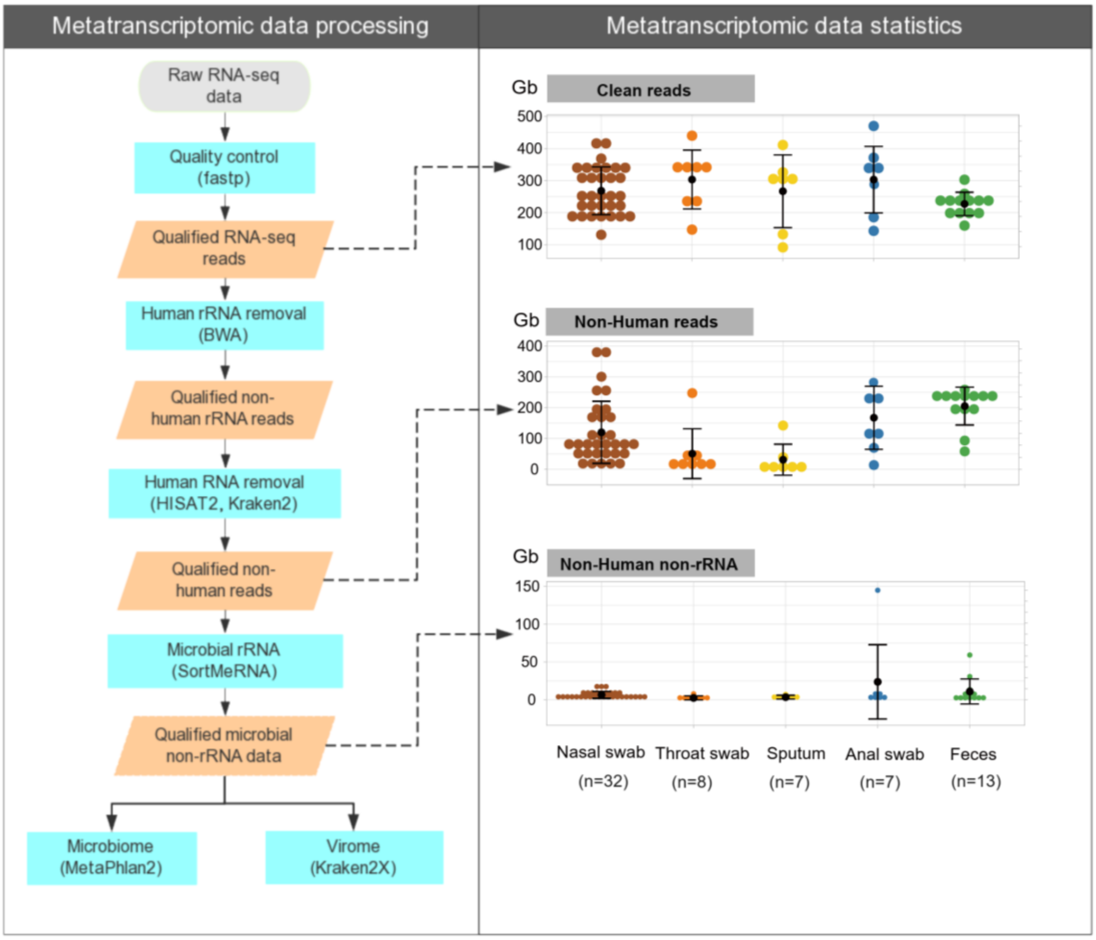

Supplementary Fig. S1: Flowchart of the metatranscriptomic analysis pipeline

To capture as much COVID-19 information as possible, total RNA was extracted from patients' clinical samples and subjected to metatranscriptomic sequencing without removing ribosomal RNA (rRNA). Raw metatranscriptomic sequencing data from all clinical samples were processed as follows: 1) quality control by fastp and generation of clean data, 2) identification and removal of human rRNA from high-quality clean data by BWA, 3) identification and removal of human non-rRNA reads by HISAT2(hg19) and Kraken2 (hg38) and generation of non-human RNA-seq reads, 4) identification and removal of microbial rRNA reads by SortMeRNA and generation of non-human non-microbial rRNA RNA data for subsequent analyses of virome (by Kraken2X) and non-viral microbiome (by MetaPhlan2). To ensure sufficient metatranscriptomic sequencing depth, samples with less than 1 million paired-end non-human, non-microbial RNA reads were excluded. As a result, 67 datasets from the 23 patients passed the criterion were included in this study. Total clean RNA-seq data (upper panel), total non-human RNA-seq data (middle panel) and total non-human non-rRNA microbial RNA-seq data (lower panel) of 67 clinical samples are shown. Brown, throat swab (n=32); orange, nasal swab (n=8); yellow, sputum(n=7); blue, anal swab(n=7); and green, feces (n=13). Detailed statistics for each processing step are shown in **Supplementary Table S3.**

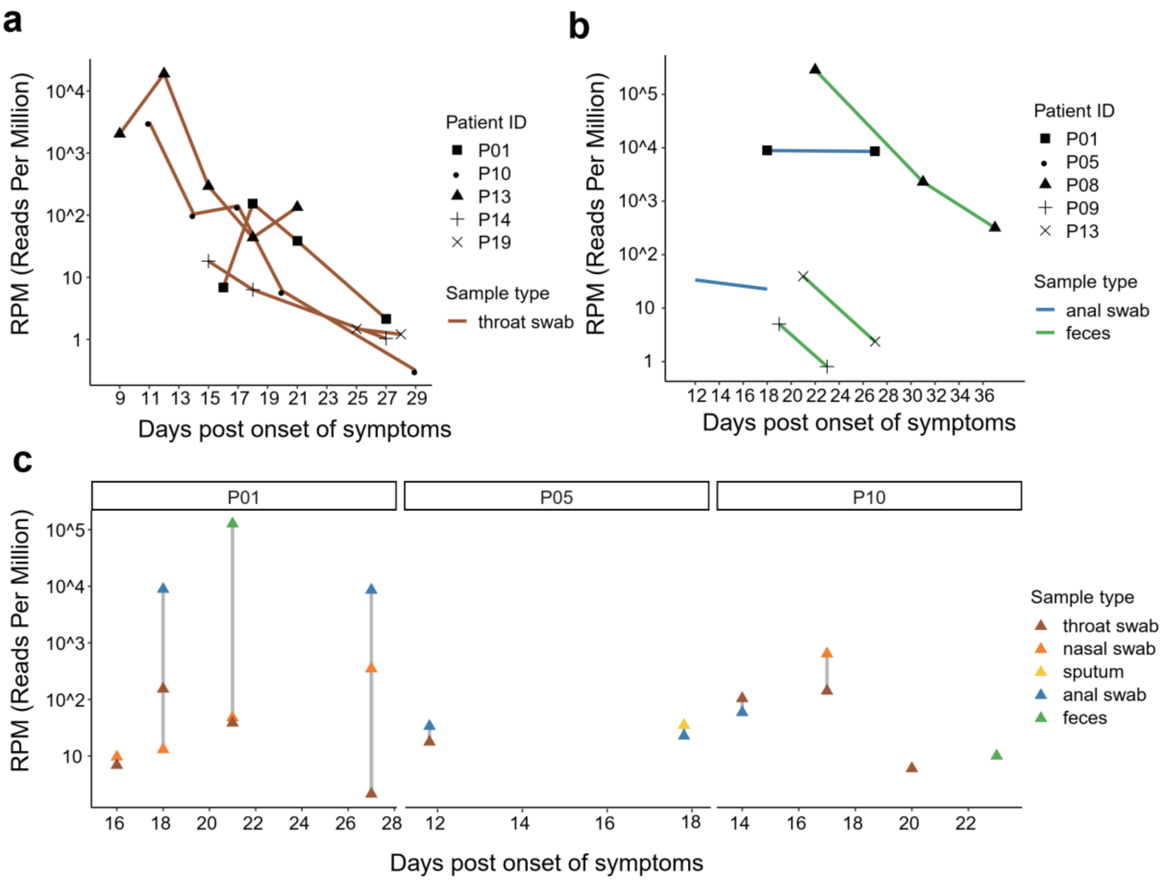

Supplementary Fig. S2: Distribution of SARS-CoV-2-RPM across specimens and individuals

a-b, Individual-based changes in the number of SARS-CoV-2-like reads in specimens from the respiratory tract (**a**) and the gastrointestinal tract (**b**). The x-axis indicates the days post-onset of symptoms (**Supplementary Table S1**).

c, Individual-based patterns in the number of SARS-CoV-2-like reads in different types of clinical specimens. Data have been normalized to total sequencing reads in reads-per-million (RPM). Different sample types are colored as follows: brown, throat swab; orange, nasal swab; yellow, sputum; blue, anal swab; green, feces. Gray, number of non-*Coronaviridae* viral reads.

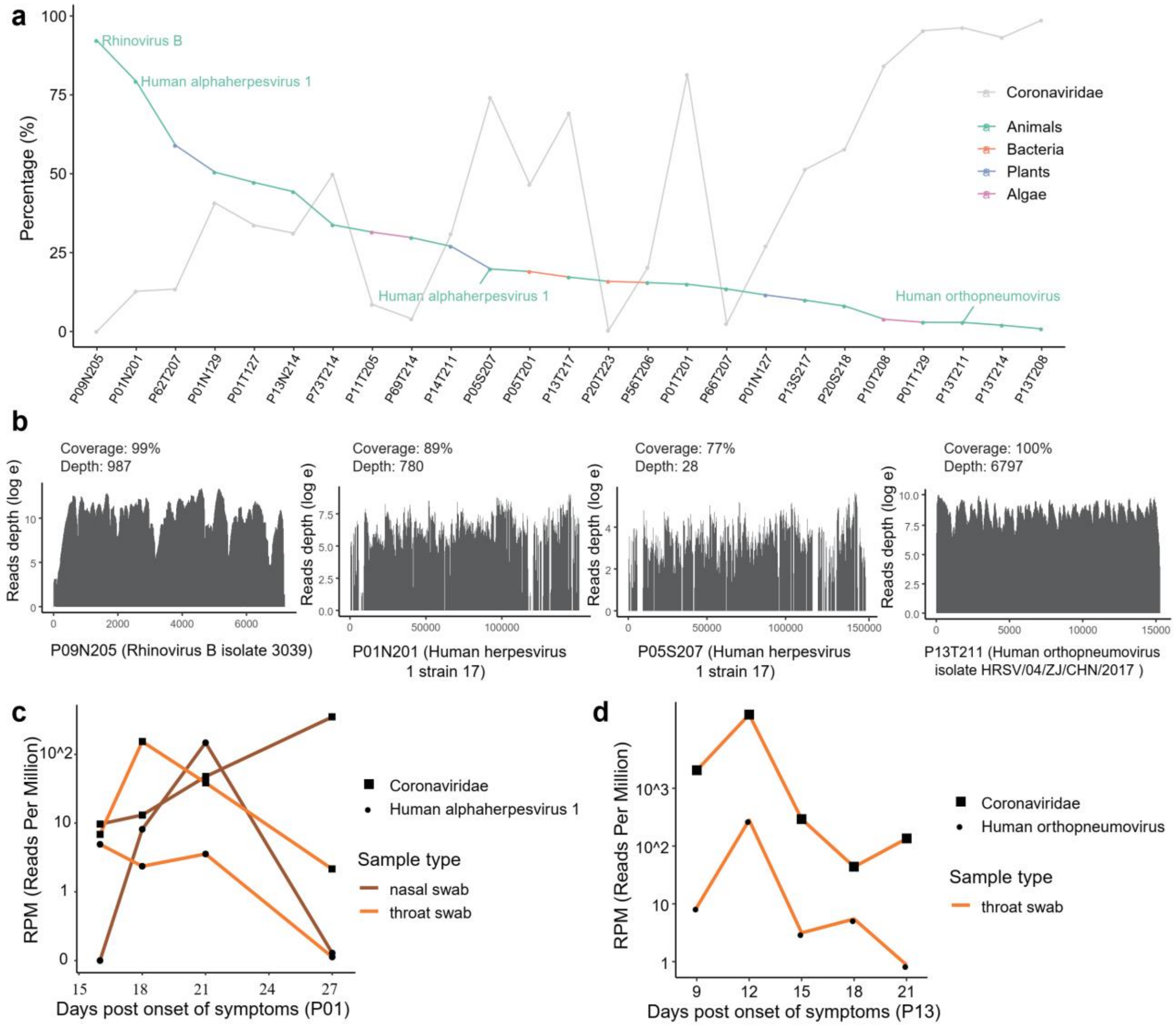

Supplementary Fig. S3: Highly abundant non-SARS-CoV-2 viruses identified in respiratory tract specimens

a, Percentage of *Coronaviridae* reads and highly abundant non-SARS-CoV-2 viral species to the total viral reads. Only non-SARS-CoV-2 viruses with >10,000 mapped reads and ranked in top 1 of all non-SARS-CoV-2 viruses are shown (See details in **Supplementary Table S5**). Viral species are colored according to their natural hosts: green, animals; pink, bacteria; light blue, plant; and purple, algae.

b, Depth and coverage of known respiratory viruses in four severely ill patients. Four representative specimens collected from four severely ill patients are robustly co-detected (genome coverage>50%) with rhinovirus B (P09N205), human herpesvirus 1 (P01N201 and P05S207) and human orthopneumovirus (P13T211).

c-d, Individual-based changes in the number of *Coronaviridae* reads and other known respiratory viruses in respiratory tract specimens from P01 (**c**) and P13 (**d**). The x-axis indicates the days post-onset of symptoms. Data have been normalized to total sequencing reads in reads-per-million (RPM).

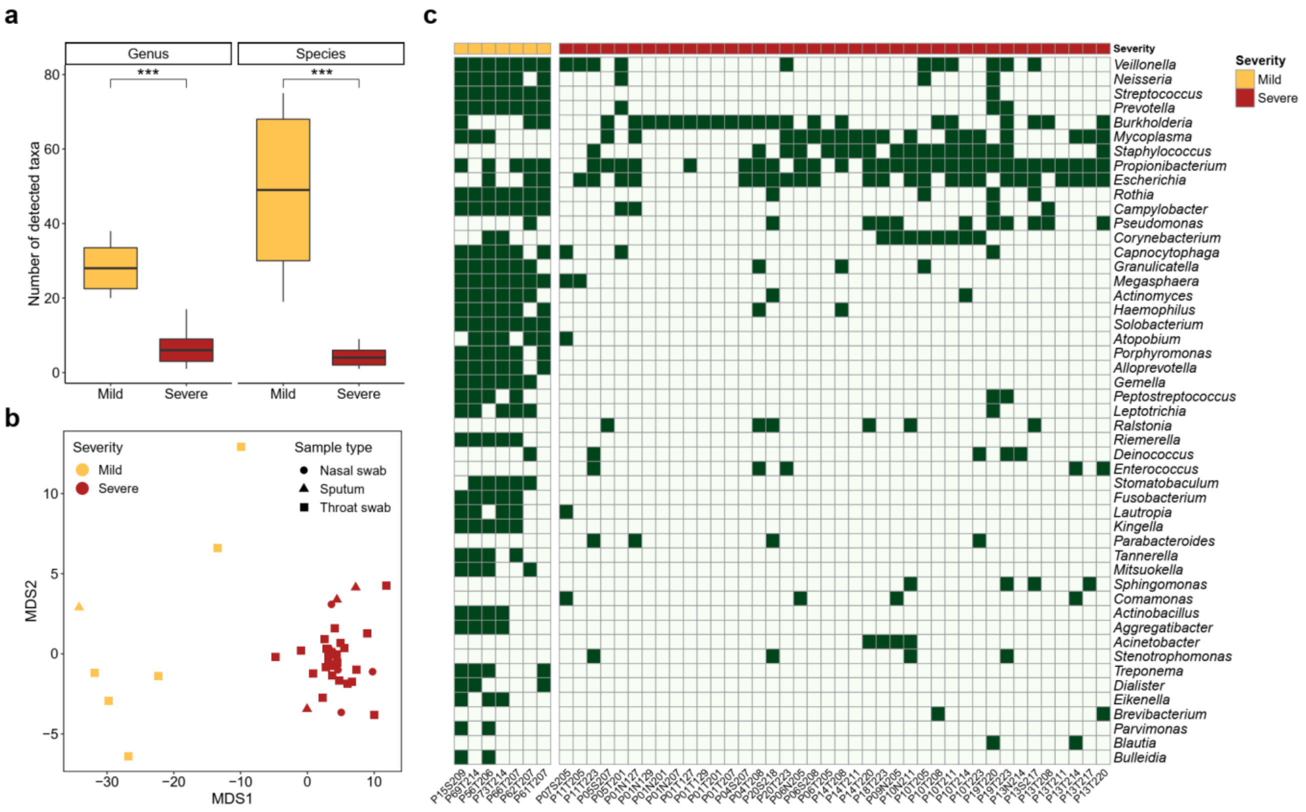

Supplementary Fig. S4: Distinct respiratory microbial signatures in mildly and severely ill patients

a, Number of detected non-viral microbial genus and species in all respiratory specimens of mild (orange, n=7) and severe cases (brick red, n=40). ***, $P < 0.001$, Wilcoxon rank-sum test.

b, Non-metric multidimensional scaling (NMDS) plot displaying distinct respiratory microbial communities in specimens from mild (orange) and severe (brick red) cases. Different shapes indicate different sampling sites: circles for nasal swabs; triangles for sputum and squares for throat swabs. Manhattan distances based on presence/absence profile at the genus level are used for NMDS ordination.

c, Presence/absence profile of non-viral microbial genera in all respiratory specimens. Only genera presented in at least two samples are shown.

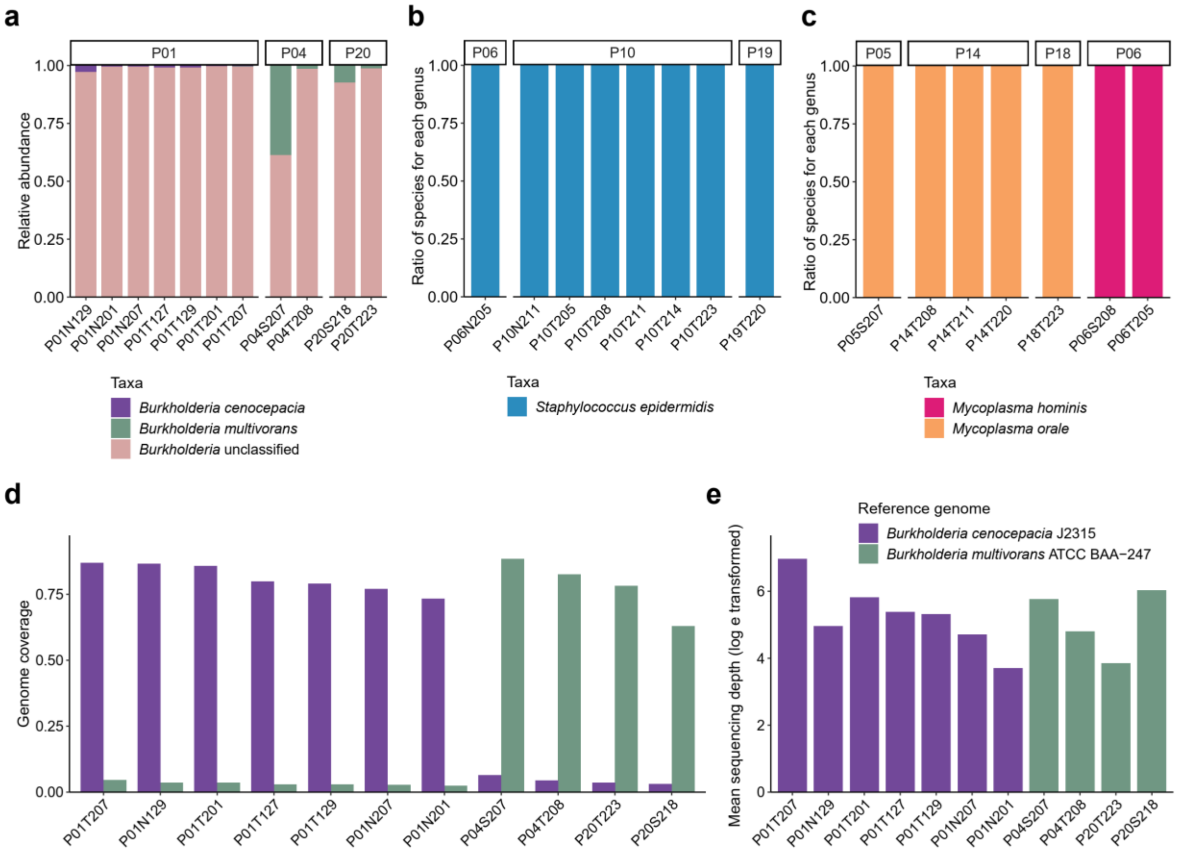

Supplementary Fig. S5: Species-level identification of genera *Burkholderia*, *Staphylococcus* and *Mycoplasma*

a-c, Bar plot showing the ratio of assigned species within *Burkholderia* (**a**), *Staphylococcus* (**b**) and *Mycoplasma* (**c**) by MetaPhlAn2. Only respiratory samples with mono-dominance of one single genus (relative abundance >60%) are shown.

d, Average coverage of two references of *Burkholderia cepacia* complex. References are shown if 10% or more of the reference genome are covered by reads in at least one sample (**Supplementary Table S8**).

e, Average sequencing depth (log e-transformed) of the two *Burkholderia* references. Purple, *Burkholderia cenocepacia* J2315, green, *Burkholderia multivorans* ATCC BAA-247.

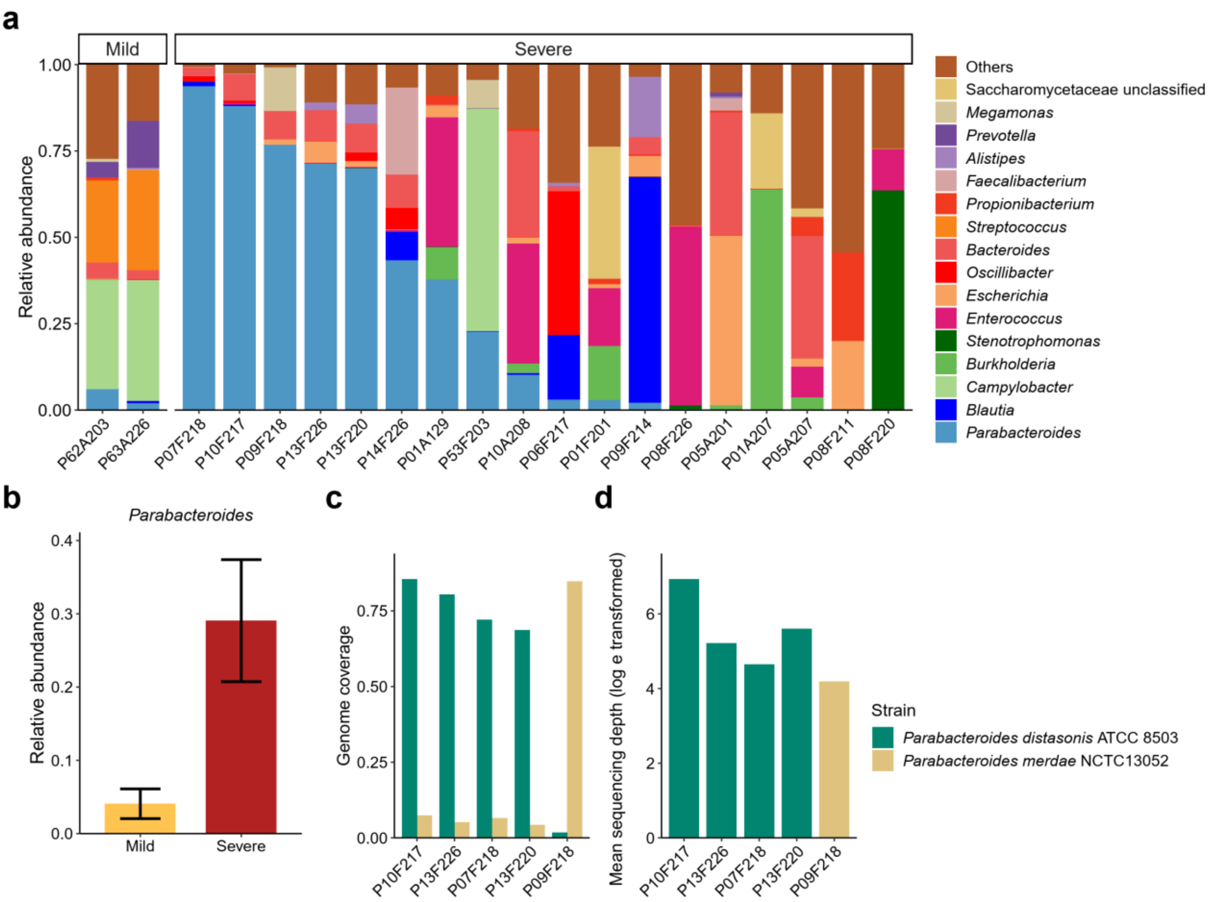

Supplementary Fig. S6: Gut microbial signatures in mildly and severely ill patients

a, Bar plot showing the relative expression levels of non-viral microbes in all gastrointestinal tract specimens.

b, Relative expression levels of *Parabacteroides* between gut specimens from mild and severe cases. The bar chart and black error bars denote the mean and standard error values of expression levels in mild (orange) and severe (brick red) cases.

c, Average coverage of two references of genus *Parabacteroides*. References are shown if 10% or more of the reference genome are covered by reads in at least one sample (**Supplementary Table S8**).

d, Average sequencing depth (log e-transformed) of the two *Parabacteroides* references. Green, *Parabacteroides distasonis* ATCC 8503*,* yellow, *Parabacteroides merdae* NCTC13052.
